# Deep Learning Using Multiple Degrees of Maximum-Intensity Projection for PET/CT Image Classification in Breast Cancer

**DOI:** 10.3390/tomography8010011

**Published:** 2022-01-05

**Authors:** Kanae Takahashi, Tomoyuki Fujioka, Jun Oyama, Mio Mori, Emi Yamaga, Yuka Yashima, Tomoki Imokawa, Atsushi Hayashi, Yu Kujiraoka, Junichi Tsuchiya, Goshi Oda, Tsuyoshi Nakagawa, Ukihide Tateishi

**Affiliations:** 1 Department of Radiology, Tokyo Medical and Dental University, Tokyo 113-8510, Japan; k.veg1589@gmail.com (K.T.); junoym@gmail.com (J.O.); m_mori_116@yahoo.co.jp (M.M.); ymgdrnm@tmd.ac.jp (E.Y.); 11.ruby.89@gmail.com (Y.Y.); toimokawa@gmail.com (T.I.); junjiatsushi@gmail.com (A.H.); kujira_cyclotol@hotmail.com (Y.K.); tuwu11@gmail.com (J.T.); ttisdrnm@tmd.ac.jp (U.T.); 2 Department of Surgery, Breast Surgery, Tokyo Medical and Dental University, Tokyo 113-8510, Japan; odasrg2@tmd.ac.jp (G.O.); nakagawa.srg2@tmd.ac.jp (T.N.)

**Keywords:** breast cancer, PET, image classification, deep learning, convolutional neural network

## Abstract

Deep learning (DL) has become a remarkably powerful tool for image processing recently. However, the usefulness of DL in positron emission tomography (PET)/computed tomography (CT) for breast cancer (BC) has been insufficiently studied. This study investigated whether a DL model using images with multiple degrees of PET maximum-intensity projection (MIP) images contributes to increase diagnostic accuracy for PET/CT image classification in BC. We retrospectively gathered 400 images of 200 BC and 200 non-BC patients for training data. For each image, we obtained PET MIP images with four different degrees (0°, 30°, 60°, 90°) and made two DL models using Xception. One DL model diagnosed BC with only 0-degree MIP and the other used four different degrees. After training phases, our DL models analyzed test data including 50 BC and 50 non-BC patients. Five radiologists interpreted these test data. Sensitivity, specificity, and area under the receiver operating characteristic curve (AUC) were calculated. Our 4-degree model, 0-degree model, and radiologists had a sensitivity of 96%, 82%, and 80–98% and a specificity of 80%, 88%, and 76–92%, respectively. Our 4-degree model had equal or better diagnostic performance compared with that of the radiologists (AUC = 0.936 and 0.872–0.967, *p* = 0.036–0.405). A DL model similar to our 4-degree model may lead to help radiologists in their diagnostic work in the future.

## 1. Introduction

Breast cancer (BC) is the most common cancer and the second leading cause of cancer-related deaths among women, and its incidence has increased recently [1]. Fluorine-18-fluorodeoxyglucose (^18^F-FDG)-positron emission tomography (PET)/computed tomography (CT) is mainly used to search for distant metastases and secondary cancers, perform staging, and monitor the response to therapy [2,3,4].

However, ^18^F-FDG-PET/CT is accurate for staging and assessing treatment response in a variety of malignancies [2,5]. Indeed, ^18^F-FDG-PET/CT is routinely used to image the entire body, at least from the mid-orbit to the proximal thigh, including the entire thorax and breast tissue. This imaging has led to incidental detection of other primary malignancies, including BC. For example, Benveniste et al. [6] reported that 440 incidental breast lesions were identified in 1951 patients who underwent ^18^F-FDG-PET/CT.

Deep learning (DL) algorithms are rapidly increasing in their use for medical imaging applications [7]. Convolutional neural network (CNN), one of the DL algorithms, has shown excellent performance in recent years for medical image processing, such as for pattern recognition, segmentation, object detection, and image synthesis [8,9,10,11,12]. In the domain of breast imaging, DL methods have been used to detect bone or lymph node metastasis [13,14] or to pathologically distinguish features between BC and normal tissue [15]. To the best of our knowledge, few studies have investigated the detection of a primary lesion in BC on ^18^F-FDG-PET/CT by using DL methods.

This study sought to determine whether a DL model can classify maximum-intensity projection (MIP) images of ^18^F-FDG-PET which consist of projecting the voxel with the highest FDG uptake value on every view throughout the volume onto a 2-dimension image into two categories, with BC or without BC, and to compare the diagnostic ability of these models with that of radiologists.

## 2. Materials and Methods

### 2.1. Patients

Selection criteria for patient enrollment in the study were: (1) 250 female patients with pathologically confirmed BC, and (2) the same number of female patients without breast disease, including BC, who underwent ^18^F-FDG-PET/CT at our hospital between April 2017 and March 2021. Exclusion criteria were: (1) history of breast resection, (2) treatment with hormonal therapy, chemotherapy, or radiotherapy for BC, and (3) age younger than 20 years. We obtained digital imaging and communications in medicine (DICOM) images for these 500 patients: 250 had BC confirmed pathologically by biopsy or surgery, and the other 250 had no BC and no history of breast disease or abnormal uptake of ^18^F-FDG-PET/CT in the chest, including the breast.

### 2.2. PET/CT Protocols

All patients were intravenously administered ^18^F-FDG (3.7 MBq/kg; 0.1 mCi/kg) after at least a 4-h fasting period. Next, whole-body images were obtained routinely using 3 different PET/CT systems: 45 cases by Aquiduo (Toshiba Medical Systems, Tokyo, Japan), 232 cases by Celesteion (Canon Medical Systems, Tochigi, Japan), and 223 cases by Cartesion Prime (Canon Medical Systems, Tochigi, Japan). In addition, CT was performed using the following parameters: pitch, 0.938; gantry rotation time, 0.5 s; table time, 30 mm/s; automatic exposure control (SD 20), 120 kV; and slice thickness, 2.0 mm. Notably, contrast materials were not used for CT examinations. After approximately 60 min of ^18^F-FDG administration, whole-body emission PET was performed using the following parameters: Aquiduo—emission time per bed, 2 min; bed positions, 7–8; slice thickness, 3.375 mm; and matrix, 128 × 128, Celesteion—emission time per bed, 2 min; bed positions, 9–10; slice thickness, 4.08 mm; and matrix, 144 × 144, and Cartesion Prime—emission time per bed, 90 sec; bed positions, 6–7; slice thickness, 2.00 mm; and matrix, 336 × 336. Both Celesteion and Cartesion Prime use the time-of-flight method that improves the signal-to-noise ratio of PET images and increases the standardized uptake value (SUV); however, Aquiduo does not use that method [2].

### 2.3. Data Set

For each patient, we obtained MIP images with 4 different degrees (0°, 30°, 60°, 90°). Table 1 summarizes the number of images and the clinical T categories according to the TNM classification 8th edition. First, we randomly split the image data into training, validation, and test image sets. For the training and validation phase, we used 400 sets of MIP images (200 BC, 200 non-BC) and labeled them into 2 classes according to the existence of BC. For the test phase, 100 sets of MIP images (50 BC, 50 non-BC) were used. The data used in the test phase were independent and were not used in the training or validation phases.

### 2.4. Image Processing

The image sets were further processed and augmented by using code written in the programming language Python 3.7.0 (accessed on 21 July 2021 https://www.python.org) and Python imaging library of Pillow 3.3.1 (accessed on 21 July 2021 https://pypi.python.org/pypi/Pillow/3.3.1). Image processing was performed separately for the training, validation, and test image sets.

For the training image sets, image processing that cut out the top and bottom of each image, approximately corresponding to the brain and bladder, and data augmentation were performed such that the CNN model became robust against the degree of enlargement, rotation, changing brightness and contrast, horizontal flip, and partial lack of image. Through those processes, 16 image sets were generated from one image set, resulting in a total of 5120 image sets (320 image sets of each phase of 5-fold cross validation × 16) that were available for training use. For each validation and test image set, like the training phase set, the top and bottom of each image (approximately corresponding brain and bladder) were cut out at first, and the central part (299 × 299 pixels) of captured images was cropped.

### 2.5. DL Methods

We performed the whole process using a computer with a GeForce RTX 2080Ti (NVIDIA, Santa Clara, California, CA, USA) graphics processing unit, a Core i7-10700 K 3.80-GHz (Intel, Santa Clara) central processing unit, and 32 GB of random-access memory. The Python programming language and Pytorch 1.6.0 (accessed on 24 July 2021 https://pytorch.org/) framework for neural networks were used for building DL models.

**Figure 1:**
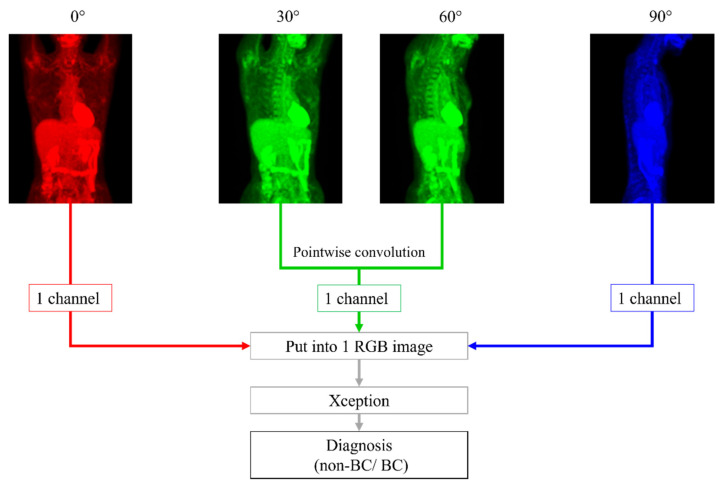
With 30° and 60°positron emission tomography (PET) maximum-intensity projection (MIP) images, pointwise (1 × 1) convolution was performed first. We placed 0°, 30° + 60°, and 90° PET MIP images into an RGB image with 3 channels: red channel for a 0° PET MIP image, green channel for a 30° + 60° image, and blue channel for a 90° PET MIP image. Next, breast cancer was diagnosed with this RGB image using Xception.

We made 2 DL models based on Xception, architecture of which has 36 convolutional layers forming the feature extraction base of the network [16]. One model, named the *0-degree model*, diagnosed BC with only 0° PET MIP image. The other was a model using 4 different degrees of images: 0°, 30°, 60° and 90° PET MIP images, named the *4-degree model*. First, pointwise (1 × 1) convolution was performed with 30° and 60° images, and a 30° + 60° image was created. Second, the 0° PET MIP image, 30° + 60° image, and 90° PET MIP image were placed into an RGB image with 3 channels: the red channel for 0° PET MIP image, green channel for 30° + 60° image, and blue channel for 90° PET MIP image. Then, BC was diagnosed with the RGB image by Xception. Pointwise convolution is a type of convolution method that uses a 1 × 1 kernel, which iterates through every single point [17]. This method makes the channels for the input images reduce and makes multiple images train at the same time. In addition, it can reduce the computational complexity of DL models [18]. By using this technique, we could input 4 images, including 0°, 30°, 60°, and 90° MIP, into Xception that needs images composed of 3 channels (Figure 1).

For training, image sets were prepared as described previously in the image processing section and were provided to each CNN. The output data were compared with the teacher data (2 categories: BC or non-BC), and the error was back-propagated to update parameters in each CNN so that the error between the output data and teacher data would be minimal. The CNNs comprised several layers, including convolutional layers, are popular for image recognition.

The CNNs were initialized by the ImageNet (accessed on 24 July 2021 http://www.image-net.org/) pretraining model and fine-tuned to yield better performance. The parameters of optimization were as follows: optimizer algorithm = stochastic gradient descent, learning rate = 0.0001 which is scheduled to decay by 0.4 every 15 epochs, weight decay = 0.001, and momentum = 0.9. The image sets for the training and validation phase were randomly split into training data and validation data at the ratio of 4:1 in each fold, and supervised learning by 30 epochs was performed.

After developing models, we tested them with more image sets that included 50 BC patients and 50 non-BC patients.

### 2.6. Radiologists’ Readout

For this study, 5 radiologists assessed the data with the following years of experience: Readers 1 and 2 had 1 year of experience, Reader 3 had 11 years, Reader 4 had 9 years, and Reader 5 had 8 years of experience in breast imaging. These 5 radiologists blindly evaluated the possibility of existence of BC (0–100 %) in 0°, 30°, 60°, and 90° MIP DICOM images of the test cases. The radiologists could not refer to the original PET/CT data. None of these images were processed by cutting out the top and bottom of the image as we performed for the DL training, validation, and test phases.

### 2.7. Statistical Analysis

All statistical analysis in this study was performed using the EZR software package, version 1.54 (Saitama Medical Center, Jichi Medical University, Saitama, Japan) [19].

Interobserver agreement was assessed using the Pearson correlation coefficient and was interpreted as follows: r = 0, no linear relationship; 0 < r < 1, a positive linear trend; r = 1, a perfect positive linear trend; −1 < r < 0, a negative linear trend; and r = −1, a perfect negative trend [20]. Receiver operating characteristic (ROC) analyses were performed to calculate the area under the ROC (AUC) for performance of the CNN models and the 2 readers in probability of the existence of BC (%), respectively. An optimal cut-off value that was closest to the upper left corner was derived (the cut-off value with the highest sum of sensitivity and specificity). We performed a DeLong test to compare AUC [21]. A *p*-value of <0.05 was considered to be statistically significant.

## 3. Results

Table 2 summarizes the interobserver agreement of our 4-degree model, 0-degree model, and radiologists.

**Figure 2:**
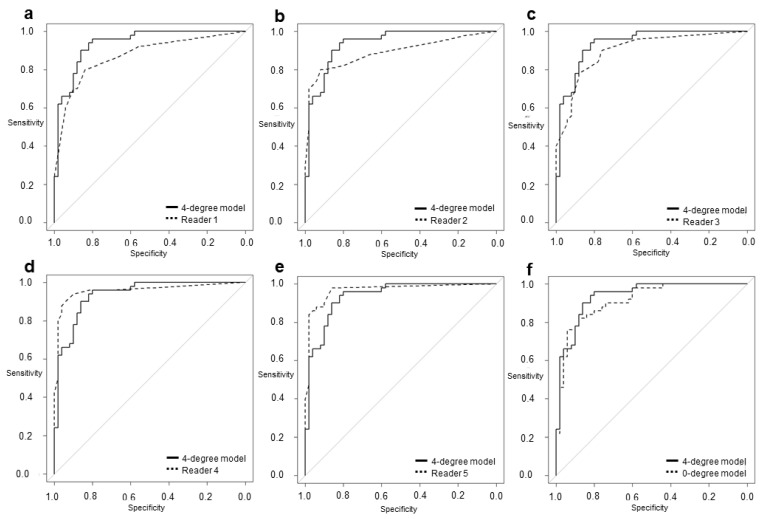
The area under the receiver operating characteristic (ROC) curve of the 4-degree model was (**a**) significantly larger than that of Reader 1 (0.936 vs. 0.872; *p* = 0.0355). The model was (**b**) not more significant but larger than that of Reader 2 (0.936 vs. 0.891; *p* = 0.189) and (**c**) Reader 3 (0.936 vs. 0.900; *p* = 0.322) and was (**d**) smaller but not significantly different from that of Reader 4 (0.936 vs. 0.957; *p* = 0.405) and (**e**) Reader 5 (0.936 vs. 0.967; *p* = 0.237). The model was also (**f**) not more significant but larger than the 0-degree model (0.936 vs. 0.918; *p* = 0.0781).

Significant interobserver agreement was found between all CNN models and the radiologists (r = 0.563–0.896; *p* < 0.001), although the interobserver agreement between these models and the radiologists (r = 0.563–0.754) was lower than that between the radiologists alone (r = 0.708–0.896). Table 3 and Figure 2 show a comparison between the diagnostic performance of the five readers and two models.

Readers 1, 2, 3, 4, and 5 had sensitivities of 80%, 80%, 90%, 94%, and 98%; specificities of 84%, 92%, 76%, 90%, and 86%; and AUCs of 0.872, 0.891, 0.900, 0.957, and 0.967, respectively. Our 4-degree model showed a sensitivity of 96%, a specificity of 80%, and an AUC of 0.936. Our 0-degree model showed a sensitivity of 82%, a specificity of 88%, and an AUC of 0.918. The AUC of our 4-degree model was significantly larger than that of Reader 1 (0.936 vs. 0.872; *p* = 0.036). Although there was no significant difference, the AUC of the 4-degree model was larger than that of Reader 2 (0.936 vs. 0.891; *p* = 0.189), Reader 3 (0.936 vs. 0.900; *p* = 0.322), and the 0-degree model (0.936 vs. 0.918; *p* = 0.078).

**Figure 3:**
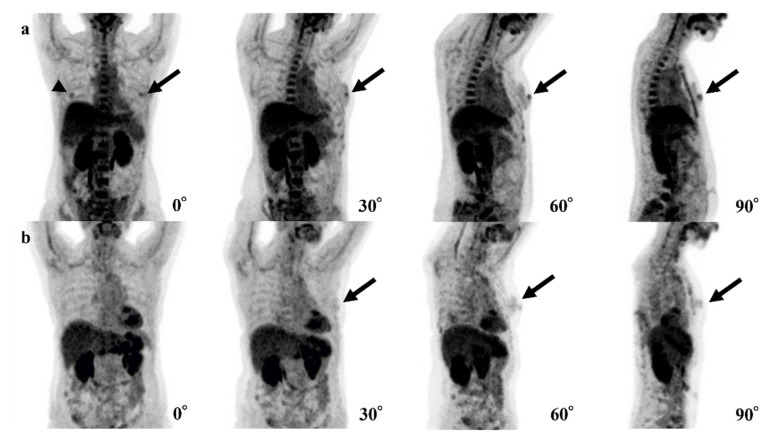
Examples of false-positive cases of the 4-degree model are shown. (**a**) The fluorodeoxyglucose (FDG) uptake of both nipples (left; black arrows, right; arrowhead) could be confirmed in the 0° positron emission tomography (PET) maximum-intensity projection (MIP) image, but the uptake of the right nipple disappears in 30°, 60°, and 90° PET MIP images. (**b**) Physiological FDG uptake of a mammary gland or a nipple (black arrows) could be recognized as a breast lesion.

**Figure 4:**
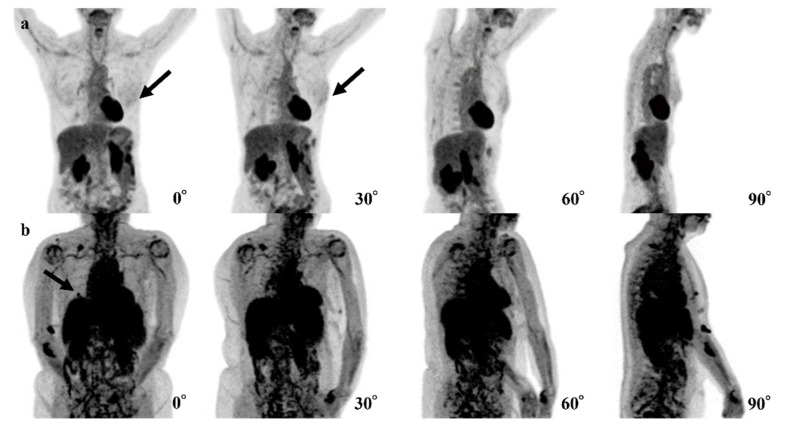
Examples of false-negative cases of the 4-degree model are shown. (**a**) The fluorodeoxyglucose (FDG) uptake at left breast cancer (black arrows) is very low and difficult to recognize. (**b**) The right breast cancer is recognizable in the 0° and 90° positron emission tomography (PET) maximum-intensity projection (MIP) images (black arrows) but is difficult to recognize in the 30° and 60° PET MIP images due to physiological FDG uptake of other organs.

In our 4-degree model, there were 10 false-positive (Figure 3) and three false-negative cases (Figure 4). Among these 10 false-positive cases, four cases had physiological FDG uptake at both (2 cases) or left (2 cases) mammary glands resembling masses; four cases had both nipples with physiological FDG uptake, but 1 of them disappeared in 30°, 60°, or 90° MIP.

Table 4 summarizes three false-negative cases. In two cases, lesions showed the maximum SUV (SUVmax) of 0.9 and 1.2. In the other case, the organs that are near the breast (heart, liver, spleen, and kidneys), showed up to SUVmax of 7.375.

**Figure 5:**
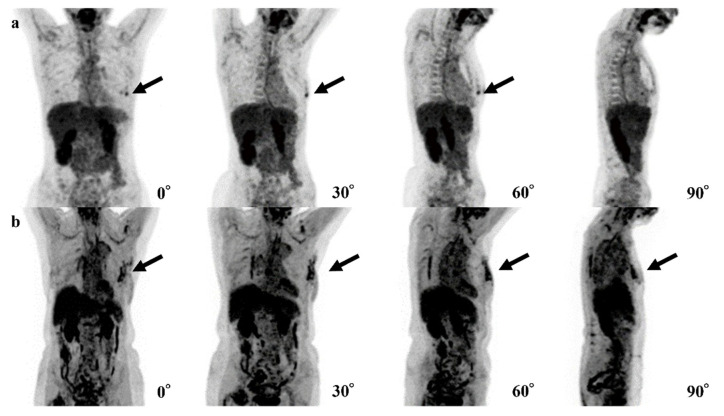
Examples of the mistakes made by the 0-degree model, for which the 4-degree model are shown. (**a**) The fluorodeoxyglucose (FDG) uptake at left breast cancer (black arrows) is very low and difficult to recognize. (**b**) The right breast cancer is recognizable in the 0° and 90° maxi-mum-intensity projections (MIPs) (black arrows) but is difficult to recognize in the 30° and 60° MIPs due to physiological FDG uptake of other organs.

In six cases, the 0-degree model made mistakes, for which the 4-degree model made the correct diagnosis. The FDG uptake of BCs was shown near the nipple in three of these cases, and the shape of FDG uptake in BC was a non-mass-like lesion in another case (Figure 5).

The DL technologies are used increasingly in the field of breast imaging such as mammography [22,23] and ultrasonography [24]. Some of these technologies (e.g., MammoScreen) support radiologists in diagnosing BC clinically. Raya-Povedano et al. [25] reported that digital mammography screening strategies based on artificial intelligence systems could reduce the workload for radiologists by up to 70%. To our knowledge, however, few software programs with MIP of PET/CT are used clinically.

The sensitivity and specificity of ^18^F-FDG-PET/CT in diagnosing primary lesion or lesions of BC by radiologists varies from 48–96% and 73–100%, respectively [4]. The increase in ^18^F-FDG-PET/CT use may lead to an increased possibility of detecting incidental breast abnormality. The use of MIP in ^18^F-FDG-PET/CT allows the clinician to easily view the whole body; therefore, it is also useful in screening for breast abnormality.

Our research focused on detecting primary BCs on MIP of ^18^F-FDG-PET/CT using several DL methods with CNNs to evaluate their diagnostic performance compared with human readers. To our knowledge, this study is the first to compare the diagnostic performance of classifying primary lesions of BC among two CNN models and human readers on MIP of ^18^F-FDG-PET/CT.

Our 4-degree model showed significantly better results in diagnosing primary BC than one less-experienced radiologist, and, although not significantly different, this model also showed better diagnostic performance than another less-experienced radiologist and one expert radiologist. In addition, no significant differences were found between the model and two expert radiologists. Based on these results, a DL model like our 4-degree model may decrease the occurrence of overlooking an incidental but critical breast abnormality, especially when the model is used to support a less-experienced radiologist and to minimize the negative effect for patients.

In this study, we examined the interobserver agreement between the CNN models and radiologists and found significant interobserver agreement between them. However, the interobserver agreement between these models and the radiologists were shown to be lower than the agreement between the radiologists alone. These findings may suggest that, although the radiologists and the CNN models made similar diagnosis, they may have different decision criteria. In the future, more accurate models will be developed by visualizing and validating the CNN model and the human rationale for the decision.

Our 4-degree model also showed non-significant but better diagnostic performance than the 0-degree model. In fact, six cases including a non-mass-like lesion were diagnosed correctly only by the 4-degree model. Hosni et al. [26] reported that ensemble methods, a technique that combines a set of single techniques, show better performance in breast image classification. Nobashi et al. [27] also demonstrated that CNNs with the ensemble of multiple images of different axes and window settings improved performance over the models using single image in the domain of brain ^18^F-FDG-PET scans. Considering the findings of these reports and our results, using multiple images may contribute to an increase in diagnostic performance more than using only one image.

In 4 of 10 false-positive cases of the 4-degree model, it is possible that our 4-degree model misrecognized normal FDG uptake of one nipple as BC. Because FDG uptake of the heart is typically higher than that of nipples, it is considered that the model could not recognize one nipple overlapping with the heart and presume the other nipple was the breast abnormality (Figure 4a). In the other four cases, the model may have misrecognized normal but mass-like FDG uptake of a mammary gland or a nipple as a breast lesion (Figure 4b).

For the three false-negative cases, it is possible that the level of FDG uptake at the lesions was insufficiently high (Figure 5a) or that the high level of physiologic FDG uptake in other organs led the model to avoid recognizing the lesions (Figure 5b). For these reasons, the model seemed not to be able to detect the abnormal FDG uptake. In two of these false-negative cases, the cancer subtypes were ductal carcinoma in situ (DCIS). The size of these lesions might be too small and low FDG uptake to recognize lesions. The remaining case was a small 8 mm invasive carcinoma with low activity and was a luminal A type.

This study has several limitations. First, sample size is small. Second, the design is a single-center and retrospective study. Third, we did not consider benign lesions such as fibroadenoma and intraductal papilloma. Forth, differences in the image quality among PET/CT devices may have influenced the diagnostic performance of our DL models. Fifth, only four types of PET MIP images were used in the construction of the DL and the radiologists’ reading. In the future, a large-scale, multicenter, prospective, validation study should be performed using a large amount of ^18^F-FDG-PET/CT data.

## 4. Conclusions

Our 4-degree model, using images that consisted of multiple degrees, was significantly more accurate than the diagnosis of an inexperienced radiologist and was comparable to that of three expert radiologists and the 0-degree model. Therefore, a DL model similar to our 4-degree model may lead to a decrease in missing incidental breast findings and may help radiologists in their diagnostic work in the future.

## Figures and Tables

**Table 1:** Number of images per clinical T categories in the training and test data.

Clinical T Category		Diameter of Invasion (x cm)	Training Data (n)	Test Data (n)
T1	T1a	0.1 < x ≤ 0.5	2	0
	T1b	0.5 < x ≤ 1.0	18	3
	T1c	1.0 < x ≤ 2.0	130	27
T2		2.0 < x ≤ 5.0	44	19
T3		5.0 < x	6	4
Total			200	50

**Table 2:** Interobserver agreement.

	Reader 1	Reader 2	Reader 3	Reader 4	Reader 5	0-deg	4-deg
Reader 1	1	0.823	0.708	0.803	0.733	0.713	0.754
Reader 2	0.823	1	0.786	0.896	0.823	0.693	0.6937
Reader 3	0.708	0.786	1	0.754	0.723	0.581	0.563
Reader 4	0.803	0.896	0.754	1	0.896	0.718	0.741
Reader 5	0.733	0.823	0.723	0.896	1	0.682	0.709
0-deg	0.713	0.693	0.581	0.718	0.682	1	0.910
4-deg	0.754	0.697	0.563	0.742	0.709	0.910	1
	Comparison was performed with the Pearson product-moment correlation coefficient. All interobserver agreements were significant (*p* < 0.001).						

0-deg: 0-degree model, 4-deg: 4-degree model.

**Table 3:** Comparison between the diagnostic performance of deep learning models and radiologists.

Model or Radiologist	Cut-off	Sp	Sn	AUC	95% CI	*p* Value
4-degree model	0.52	0.80	0.96	0.936	0.890–0.982	—
0-degree model	0.51	0.88	0.82	0.918	0.859–0.968	0.078
Reader 1	0.50	0.84	0.80	0.872	0.804–0.941	0.036
Reader 2	0.40	0.92	0.80	0.891	0.824–0.957	0.189
Reader 3	0.10	0.76	0.90	0.900	0.841–0.960	0.332
Reader 4	0.20	0.90	0.94	0.957	0.916–0.999	0.405
Reader 5	0.10	0.86	0.98	0.967	0.934–1.000	0.237

Sp: Specificity, Sn: Sensitivity, AUC: Area under the curve, CI: Confidential interval.

**Table 4:** Summary of false-negative cases.

Case	Age	SUVmax	Breast Density	Size of Invasive Components (mm)	Total Tumor Size (mm)	Pathology and Subtype	ER	PgR	HER2	Ki67
1	44	2.0	Heterogeneously	11	11	IDC	+	+	-	9.1%
2	70	0.9	Scattered	None	0.6	DCIS	+	-	+	15.4%
3	70	1.2	Heterogeneously	None	8	DCIS	+	+	+	12.0%

ER: Estrogen receptor, DCIS: Ductal carcinoma in situ, HER2: Human epidermal growth factor type 2, IDC: Invasive ductal carcinoma, PgR: Progesterone receptor.

## Data Availability

The data presented in this study are available within this article.
